# Don't Stop Me Now, `Cause I'm Having a Good Time Screening: Evaluation of Stopping Methods for Safe Use of Priority Screening in Systematic Reviews

**DOI:** 10.1002/cesm.70068

**Published:** 2026-01-21

**Authors:** Tim Repke, Francesca Tinsdeall, Diana Danilenko, Sergio Graziosi, Finn Müller‐Hansen, Lena Schmidt, James Thomas, Gert van Valkenhoef

**Affiliations:** ^1^ Climate Economics and Policy Potsdam Institute for Climate Impact Research (PIK), Member of the Leibniz Association Potsdam Germany; ^2^ Evidence for Policy and Practice Information Center, UCL Social Research Institute, Institute of Education University College London London UK; ^3^ Centre for Clinical Brain Sciences University of Edinburgh Edinburgh UK; ^4^ NIHR Innovation Observatory Newcastle University Newcastle upon Tyne UK; ^5^ Central Executive Team Cochrane Collaboration London UK

**Keywords:** digital evidence synthesis, priority screening, stopping methods, systematic maps, systematic reviews, technology‐assisted reviews

## Abstract

**Introduction:**

Priority screening has the potential to reduce the number of records that need to be annotated in systematic literature reviews. So‐called technology‐assisted reviews (TAR) use machine‐learning with prior include/exclude annotations to continuously rank unseen records by their predicted relevance to find relevant records earlier. In this article, we present a systematic evaluation of methods to determine when it is safe to stop screening when using prioritization.

**Methods:**

We implement an open‐source evaluation framework that features a novel method to generate rankings and simulate priority screening processes for 81 real‐world data sets. We use these simulations to evaluate 15 statistical or rule‐based (heuristic) stopping methods, testing a range of hyperparameters for each.

**Results:**

The work‐saving potential and performance of stopping criteria heavily rely on “good” rankings, which are typically not achieved by a single ranking algorithm across the entire screening process. Our evaluation shows that almost all existing stopping methods either fail to reliably stop without missing relevant records or fail to utilize the full potential work‐savings. Only one method reliably meets the set recall target, but stops conservatively.

**Conclusions:**

Many digital evidence synthesis tools provide priority screening features that are already used in many research projects. However, the theoretical work‐savings demonstrated in retrospective simulations of prioritization can only be unlocked with safe and reproducible stopping criteria. Our results highlight the need for improved stopping methods and guidelines on how to responsibly use priority screening. We also urge screening platforms to provide indicators and authors to transparently report metrics when automating (parts of) their synthesis.

## 1 Introduction

The published scientific literature is growing at an impressive rate [1, 2, 3]. Although additional evidence may be good overall, it poses a considerable challenge for evidence synthesists, decision‐makers, and other users of evidence [4] as exhaustive identification of relevant evidence is an essential requirement for most methodologies. The sheer volume prohibits the use of conventional systematic map and review methods and requires (partial) automation [5, 6].

In this article, we focus on priority screening which is growing in popularity and has large work‐saving potential [7, 8]. Priority screening supports the process of deciding whether abstracts that were found by a database search are actually relevant for the current study. Therefore, unseen records are continuously ranked to always show potentially the most relevant records next using machine‐learning models trained on prior include/exclude annotations. However, this approach can only save work if the ranking is good and if there is a reliable criterion to decide when to stop screening because all relevant records are already found [9]. Furthermore, we need to ensure that those stopping methods are safe and robust enough to use responsibly while also being able to quantify remaining uncertainties.

To this end, we present an empirical evaluation of existing stopping methods on screening decisions from 81 systematic reviews to determine (i) which stopping methods work and under which conditions, (ii) how stopping methods are influenced by the priority ranking of unseen records, and (iii) how the choice of user‐defined hyperparameters impacts the performance of stopping methods.

There are already evaluation frameworks to (partially) address some of these research questions. For example, both Yang et al. and Bron et al. published Python libraries that each implement five stopping methods and abstract classification pipelines for ranking [10, 11]. Li et al. published a Python library as part of their proposed stopping framework that implements six stopping methods and has three ranking algorithms (BM25, logistic regression, support vector machines [SVM]) as well as CLEF and TREC data sets built in for evaluation [12].

However, these analyses are often part of the proposal of a new stopping method, use synthetic data, improperly apply competing stopping methods, or make unrealistic assumptions. Reviewing the literature, there appears to be no consensus on which stopping methods are reliable and how to validate that. Priority screening is already supported in several major digital evidence synthesis tools such as Covidence, EPPI‐Reviewer [13], NACSOS [14], Abstrackr [15], or Rayyan [16]. Only NACSOS implements a statistical stopping criterion for users to track the progress and determine when it is safe to stop. Some popular platforms do not even provide the relevant information to enable users to use external tools for computing stopping criteria.

With this work, we publish an easily extensible open‐source framework that implements a wide range of stopping methods in such a way that they can be easily reused elsewhere. Furthermore, the framework allows anyone to effortlessly run evaluations of simulations on their data sets. Here, we present the results from thousands of simulations using a wide range of stopping methods and hyperparameters combined with optimized ranking models using real‐world data sets. Our analysis shows that only one method, CMH, is safe to use, but it does not use the full work‐saving potential. The QUANT_CI method is safe to use most of the time, but stops too early in some cases or never for about half of our simulations.

Overall, we find that the vast majority of existing stopping methods are not fit for purpose for use in systematic maps or reviews. Our work offers a standardization for how to rigorously evaluate and compare stopping methods. We hope to inspire future research to improve stopping methods and for platform developers to integrate guidelines and indicators that empower the safe and responsible use of machine‐learning‐assisted screening tools.

## 2 Methods

For the evaluation of stopping methods we set up an open‐source modular and extensible evaluation framework (https://github.com/destiny-evidence/stopping-methods). Typically, priority screening is a combination of two main mechanisms. The first mechanism uses machine‐learning models trained on existing inclusion/exclusion annotations to then rank unseen records in descending order of relevance. In this way, annotators are not screening records at random but get to see the potentially most relevant first. At some point, all relevant records are found and the remaining unseen records do not need to be screened. However, determining this point without complete knowledge is not trivial, and the purpose of the second mechanism is the stopping criterion. Stopping criteria are rules that use indicators of the prior screening decisions, prediction scores of the ranking model, or other indicators to determine when it is safe to stop screening.

**Figure 1:**
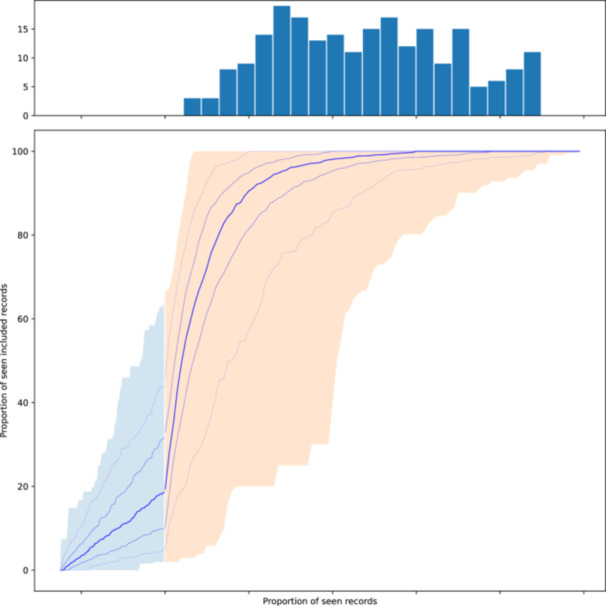
Composite of aligned inclusion curves from all screening simulations. Lines mark the mean and 5th, 25th, 75th, and 95th percentiles, and the shaded areas mark the full range. The blue area marks the initial random samples, and the orange areas mark the remainder of the data sets. The histogram depicts where a theoretical 99% recall target is reached.

Figure 1 is a composite of all inclusion curves from our simulations, also known as the gain curves, that illustrate how many included records are found as more records are screened. In an initial random sampling baseline (blue area), the slope typically follows a diagonal where relevant records are occasionally found. After switching to prioritization (orange area), most screened records are relevant, and the curve is much steeper than before and begins to flatten again once fewer relevant records remain in the unscreened set until it eventually converges.

Our framework decouples these mechanisms and simulates the screening process, including priority ranking, but without stopping. This is a computationally very expensive process and was pre‐computed on the high‐performance cluster at the Potsdam Institute for Climate Impact Research. In the second step, we test when each stopping criterion determines a safe point to stop and measure how close this is to the theoretically optimal point.

### 2.1 Priority Ranking

Conventionally, all abstracts need to be screened, and the next record to be annotated is chosen at random. Some technology‐assisted review tools use the ranking function from the retrieval system that measures how well an abstract matches the search query. A commonly used function is Okapi BM25 [43] and has some parameters that can be tuned once some data was annotated. This approach has the benefit that abstracts are ranked right from the start without the need for training data as the query serves as a prior. We do not implement BM25 or any other query‐based ranking mechanism because we do not have access to the original queries associated with the evaluation data sets. Other commonly used ranking models are based on SVMs or logistic regression models that are trained on sparse vector representations of the abstracts and existing inclusion/exclusion annotations (see, for example, AS‐review ELAS ultramodels: https://asreview.readthedocs.io/en/stable/lab/models.html). To the best of our knowledge, transformer models are not yet used in digital evidence synthesis tools or stopping method evaluations, and the use of large language models is subject to ongoing research [44, 45, 46, 47].

In regular intervals, the ranking model is retrained as additional records are annotated. Each synthesis tool offering priority screening handles this differently. Whereas some start training models right away and retrain at relatively small fixed intervals, others require a larger initial sample of records screened at random and have adaptive batch sizes. Most synthesis tools are not (fully) open‐source, but from the limited information available, there appear to be no adaptive model selection strategies or tuning.

Although the literature on technology‐assisted reviews often refers to this iterative training process as “active learning,” it is important to note that this is in fact different to what is commonly defined as “active learning” [48, 49, 50]. While there are similarities, the key difference is that in priority screening, the main purpose is to *see all* relevant records and rank the unseen records accordingly. In active learning, the goal is to build the optimal classifier with minimal annotation effort and choose previously unseen records for annotation by what is predicted to be most useful for improving the model's decision boundary.

Our pre‐computed ranking simulations use a fixed initial set of 500 randomly sampled abstracts (This value was determined experimentally, see discussion for details) after which the model is retrained in adaptive intervals. The target batch size is within a lower (25 records) and upper bound (200 records), whereas the target starts at the lower bound and is growing at a rate of 10% after each iteration. The actual batch size might be smaller or larger within the bounds if at least two new included records are found. In this way, we can reduce the computational overhead by only retraining when it is actually sensible and a sufficient amount of additional training data is available.

We implemented transformer‐based models and four basic ranking models (logistic regression, SVM, gradient descent learning, and light gradient‐boosting machine) based on sparse TF‐IDF vector representations. Each model can be used with pre‐determined hyperparameters or the hyperparameters can be optimized with Optuna [51]. Early experiments have shown that hyperparameters should be adjusted as the available set of training data is growing. Furthermore, we found that the rankings can be drastically improved by choosing the best kind of model at each retraining iteration. To this end, we use a diverse set of models with optimized hyperparameters and use prediction scores of the best‐performing model (based on calculated recall on the respective set of seen documents) to rank unseen records. Early tests have shown that the setup does not change with every retraining step, so we reused the same model and hyperparameters for four iterations. We pre‐compute three priority screening simulations per data set with a different initial random sample and different random seeds. Figure 1 shows a composite of all simulations.

### 2.2 Stopping Methods

The selection of stopping methods implemented for this evaluation as listed in Table 1 is informed by an ongoing systematic review [52]. Several methods are actually just variations of one another and may appear with different names in the literature. In general, stopping criteria can be categorized as *target‐agnostic* if the decision to stop is not based on reaching a pre‐defined metric such as recall or precision or *target‐aware* otherwise. A *target‐aware* criterion is also *uncertainty‐aware* if it offers a measure of statistical uncertainty about reaching the set target, for example, using confidence intervals or variance of an estimated recall or formal hypothesis testing procedures. Conversely, *uncertainty‐agnostic* stopping criteria solely base the decision to stop on the point estimate of the performance metric compared to the pre‐defined target. For our evaluation, we chose representative methods from each category. Where possible, we adapted existing implementations but also rewrote and adjusted some aspects of them to make them work more reliably. We iteratively apply each stopping method with a range of different hyperparameters in fixed regular intervals of 15 records on the pre‐computed ranked screening simulations. Note that these batch sizes are independent of the retraining batches from the screening simulation. Once a stopping criterion is fulfilled, we consider the end of the first batch as the stopping point for that method and that hyperparameter combination.

**Table 1:** Overview of stopping methods currently implemented in our evaluation framework and their usage in related work.

Method	Type	Hyperparameters	Proposed or evaluated in
APRIORI	Target‐agnostic	Recall threshold, window size	[10]
BATCHPRECISION	Target‐agnostic	Precision threshold, window size	[17, 18, 19]
CMH	Target‐aware, uncertainty‐aware	Recall target, confidence level	[18, 19, 20, 21, 22]
CURVE_FITTING	Target‐aware, uncertainty‐aware	Recall target, confidence level, num. windows	[23]
HEURISTIC_FIX (consecutive excludes)	Target‐agnostic	Fixed number of consecutive excludes	[20, 22, 24, 25, 26, 27, 28, 29]
HEURISTIC_FRAC (consecutive excludes)	Target‐agnostic	Fraction of consecutive excludes	[30]
HEURISTIC_RANDOM	Target‐aware, uncertainty‐agnostic	Recall target	—
HEURISTIC_SCORES/QUANT	Target‐aware, uncertainty‐agnostic	Recall target	[18, 21, 22]
IPP	Target‐aware, uncertainty‐aware	Recall target, confidence level, num. windows	[31]
KNEE	Target‐agnostic	Slope ratio, curve threshold	[12, 18, 21, 22, 23, 24, 25, 32, 33, 34, 35, 36, 37, 38, 39]
METHOD2399	Target‐agnostic	Scaling factor	[18, 19, 37]
QUANT_CI	Target‐aware, uncertainty‐aware	Recall target, confidence level	[18, 21, 22]
S‐CAL	Target‐aware, uncertainty‐agnostic	Recall target, sample size, scaling factor	[40]
${\;\text{SAL}\;}_{\tau }$	Target‐aware, uncertainty‐aware	Scaling factor, normalization, confidence level, recall target	[21]
TM_QBCB (Target method with quantile binomial confidence bound)	Target‐aware, uncertainty‐aware	Recall target, confidence level, and number of inclusion samples	[41, 42]

In the following, we explain the basic underlying principles for each of the included stopping methods. *APRIORI* [10] measures recall by comparing annotator ratings and the classifications used in the ranking and stops once the recall is above the set threshold. *BATCHPRECISION*, also known as marginal precision [17, 18], stops when the precision between classifier prediction and human annotation in the latest N screened records falls below the set threshold. *CMH* is a statistical stopping criterion that calculates the *p*‐score for the null hypothesis, that the set recall target is missed, and stops once the desired confidence target is met. [20] *CURVE_FITTING* [23] fits a negative exponential curve to the smoothed inclusion curve to estimate the total number of relevant records and derives a recall estimate from that. *HEURISTIC_RANDOM* uses the inclusion rate in the initial random sample to extrapolate the overall number of relevant records to be able to stop once an estimated recall target is reached. *HEURISTIC_SCORES*, also known as *Quant* [18], use the ranking model scores by estimating the overall number of included records and stopping at a set recall target. *QUANT_CI* [18] extends this idea by adjusting this estimation by using the variance of model scores to derive a confidence interval. *HEURISTIC_FIXED* [24] and *HEURISTIC_FRAC* [30] are commonly used methods to stop screening once a fixed number (or proportion of the data set) of consecutive excludes have been observed under the assumption that this indicates the convergence of the inclusion curve. *IPP* [31] models the actual occurrences of included records to the expected random distribution as point processes to estimate the number of expected included records. The *KNEE* method [32] is based on kneedle [33] that identifies the point where the inclusion curve begins to flatten and stops when the ratio of the slope before and after that point is above a certain threshold. In the original implementation, this worked very unreliably, so we changed the curve smoothing mechanism by fitting configurable polynomials and introducing a minimum threshold for the distance between the inclusion curve and the diagonal connecting the start and endpoint. The *2399 METHOD* [32] is triggered once the number of screened records is above at least 2399, excluding the number of included records found so far, adjusted by a factor. *S‐CAL* [40] uses ranking scores of sub‐samples of the training batches of the prioritization loop to estimate the current recall. ${\;\text{SAL}\;}_{\tau }$ [21] is an adaptation of an active learning strategy that uses the rate of change and the sum of adjusted prediction scores between iterations. There are several variations of the target method which assume some level of prior knowledge, in that known relevant records are hidden within the screening process to estimate when a pre‐determined recall target is met [32]. Although these offer statistically sound recall estimates, they effectively require additional annotation effort before the prioritization process begins and hence substantially skews “work saved” metrics. For completeness, we include one variation of the method (QBCB) [42] under the assumption that relevant records are known ahead of time, for example, by expert knowledge.

Additional methods, such as RLStop [38], Chao's estimator [22], and confidence sequences [53], require a deeper integration with the prioritization model and are beyond the scope of this analysis. We also do not include hybrid stopping methods, for example, as suggested in the “SAFE procedure” [28].

### 2.3 Data Sets

Our evaluation is based on 81 real‐world data sets of fully manually screened abstracts for systematic reviews across various research areas that are commonly used to evaluate technology‐assisted review methods. We selected those from commonly used collections of systematic review annotations, namely from CLEF‐TAR 2017–2019 [54, 55, 56] (reviews in empirical medicine), TREC 2010, 2015, 2016 tracks on total recall and legal [57, 58, 59], the SYNERGY collection (reviews in psychology, medicine, biology, computer science, and maths) which extends the Cohen collection [60, 61], and CSMeD [62] which extends some of the previously mentioned collections and others [63, 64]. Additionally, we use unpublished data sets that were kindly provided by users of the EPPI‐reviewer platform.

For smaller data sets, the benefits of safe stopping methods at higher recall targets with good certainty are minor, and simulations on low inclusion rates would mostly depend on the capability of the ranking method, which is beyond the scope of this work. To this end, we excluded many data sets because of their size (only using data sets with at least 1000 records) or their extremely low inclusion rate (below 1%). Of the 93 remaining, we excluded another seven data sets where we were not able to retrieve at least 90% of the abstracts through OpenAlex, the Web of Science, Scopus, dimensions.ai, or PubMed.

## 3 Results

The following analysis of stopping method performance is based on 223 simulated prioritized screening runs for 81 real‐world data sets of systematic reviews or other relevant decisions (see Supporting Information S1: Appendix). On average, data sets contain 3600 records (1000–13,095; STD = 2570) with on average 167 (5%) relevant records (16–1957; STD = 240 or 0.8%–37%; STD = 5%) (see Supporting Information S1: Appendix for details). In total, we recorded 20M stopping decisions across 83,999 combinations of data set, repeated screening simulations, stopping criteria, and hyperparameters.

Figure 1 shows a composite of all inclusion curves to give an indication of the quality of rankings. Ideally, a ranking model would have a slope of one (every screened record is included) after the initialization sample until all relevant records are found, and then sharply transition to a zero‐slope. Early experiments with simple machine‐learning models have produced curves that only continue along the slope of random sampling which does not allow for any early stopping. Using our continuous optimized ensemble approach, almost all simulations offer potential work savings when paired with an optimal stopping criterion. In the first quartile, only 32% of the data set needed to be screened before reaching the 99% recall target with 49% in the second and 66% in the third quartile. Additional statistics at different recall targets are included in the Supporting Information S1: Figure S1. Another observation from early experiments is that the curves often have intermediate plateaus where no new relevant records are discovered for a while. Most stopping methods detect such “steps” as a false signal and stop too early. It is our hypothesis that this pattern is caused by the ranking model being stuck in a local minimum and that the inclusion curve is temporarily growing again as a new cluster of relevant records is found. Increasing the size of the initial random sample appears to be a good strategy to mitigate this stepping pattern.

**Figure 2:**
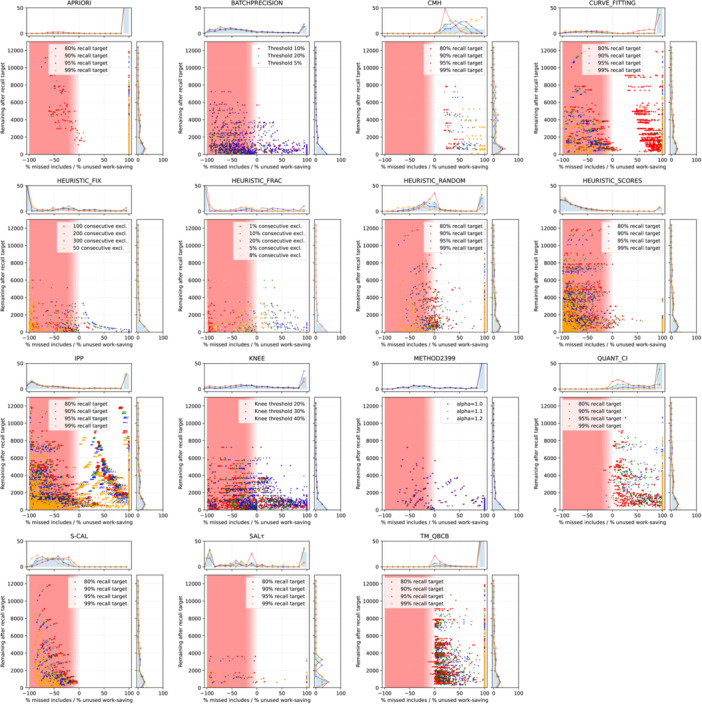
Trade‐off between work saved and missed relevant records by stopping early. The ideal stopping method would have all points along a vertical line at x = 0, where no relevant records were missed, and the stopping rule invoked at the theoretically perfect time with no additional work. Results are grouped by a dominant hyperparameter or recall target where available. The histograms show the distribution of points along each axis. Histogram lines correspond to hyperparameter groups, whereas bars show the overall distribution.

Common metrics in the literature to measure the performance of a stopping method are variations of the recall measured at the stopping point and the work saved compared to screening the complete data set. In Figure 2, we provide an overview of all results, whereas each dot represents a decision to stop screening by a method with a given set of hyperparameters for a screening simulation. We use colors and marker symbols to denote results for different recall targets where available. For methods without a target recall parameter, we group results by the most influential hyperparameter and replicate the result with different recall targets as the theoretically optimal stopping point as a reference. The *x*‐axis is split into two halves. The left half of the lower axis shows the proportion of the records that should have been included for the given recall target but were missed due to stopping too early. The right side shows what proportion of records after the optimal stopping point for a given recall target were screened until the method decided it is safe to stop, where 0% would coincide with the optimal point and 100% means the criterion was never fulfilled. This same metric is shown on the *y*‐axis as absolute values. Additional in‐depth results per method and tables listing the values of the distributions shown are available in the Supporting Information Materials.

All stopping methods, except for CMH, stop too early on many occasions. Particularly, heuristic methods that use the number of consecutive exclusions tend to miss most of the relevant records. Conversely, CURVE_FITTING, APRIORI, and recall estimation (HEURISTIC_SCORES) stop far too late or never, while still showing several cases where they stop before reaching a specific recall. Due to the nature of Method2399, it always screens 37 of our data sets completely as they contain fewer than 2399 records, but also never stops in more cases than that. BATCHPRECISION and KNEE have no clear point where they stop, far too early or late, and are spread across the entire range, independent of the hyperparameter settings. They do, however, tend to stop too early more often, the larger the data set is (Pearson's correlation: −0.46 and −0.4). The estimation of the number of included documents using the inclusion rate of the initial random sample in HEURISTIC_RANDOM tends to under‐estimate the recall target and misses records in half of all cases or almost never stops otherwise. S‐CAL consistently underestimates the expected number of relevant records and stops too early. ${\;\text{SAL}\;}_{\tau }$, for all three variants, and IPP do not appear to have a clear tendency for stopping early or late. These results indicate that these methods cannot be used to reliably determine when it is safe to stop prioritized screening.

Target methods, in our analysis, represented by the QBCB variant, estimate the current recall based on known relevant records that are hidden in the screening process. For example, a target recall of 80% requires 15 relevant records annotated ahead of time, which would require screening 40–1800 (300 on average) records at random for our evaluation data sets. We do not add the additional work required to annotate this reference data set to our metrics with the assumption that this method only makes sense for data sets with high inclusion rates or when a reference is known ahead of time. In our experiments, the TM_QBCB method tends to overshoot a lot and only stops closer to the theoretically ideal point for low recall targets with low confidence.

The QUANT_CI method uses ranking model scores to estimate a recall similar to HEURISTIC_SCORES, but also has variations to compute a confidence interval around the estimate. Doing so makes this method more conservative and overshoots the recall target by less than 20% in 26% of the cases and by less than 50% in 44% of the cases. For about 40% of the cases, more than 95% of the records after the theoretically optimal stopping point were screened, which is more pronounced for higher recall targets. In about 4% of cases, up to 50% of relevant records were missed by stopping too early.

Only one method, CMH, never stops before the set recall target. Overall, it tends to be more conservative for higher recall targets and less so for larger data sets, thus forgoing significant shares of potential work savings in these settings. This method overshoots by less than 20% in 9% of the cases and in less than 50% in 46% of the cases. Lowering the confidence level can safely reduce the overshoot by on average 23% at all recall targets.

Table 2 summarizes the results by showing the achieved recall when stopped and the respective overshoot for all recall targets (where available). It also shows the proportion of simulations where the recall target was missed.

**Table 2:** For each stopping method and target recall (if applicable), the table shows the percentage of simulations that missed the recall target, actual recall at stopping (mean over respective simulations with 10 to 90 percentile range), and additional work as percentage of the records remaining after the target recall has been reached.

Method	Recall target	% target missed	Achieved recall mean (10–90q)	Additional work (mean)
APRIORI	80	13	92 (51–100)	54
APRIORI	90	4	98 (100–100)	58
APRIORI	95	0	100 (100–100)	54
APRIORI	99	0	100 (100–100)	40
BATCHP.	—	—	76 (16–100)	—
CMH	80	0	98 (97–100)	23
CMH	90	0	99 (97–100)	28
CMH	95	0	100 (99–100)	34
CMH	99	0	100 (100–100)	37
CURVE.	80	32	76 (11–100)	35
CURVE.	90	26	82 (18–100)	40
CURVE.	95	23	84 (23–100)	37
CURVE.	99	22	85 (24–100)	22
H._FIX	—	—	34 (0–100)	—
H._FRAC	—	—	35 (0–100)	—
H._RAND.	80	45	79 (61–100)	8
H._RAND.	90	49	87 (67–100)	15
H._RAND.	95	51	89 (70–100)	14
H._RAND.	99	51	91 (73–100)	5
H._SCORES	80	91	20 (0–76)	−18
H._SCORES	90	91	23 (0–85)	−22
H._SCORES	95	91	24 (0–89)	−28
H._SCORES	99	92	25 (0–90)	−43
IPP	80	58	52 (2–100)	10
IPP	90	59	53 (2–100)	6
IPP	95	60	53 (2–100)	−0
IPP	99	62	53 (2–100)	−15
KNEE	—	—	90 (36–100)	—
M.2399	—	—	99 (96–100)	—
QUANT_CI	80	3	96 (90–100)	25
QUANT_CI	90	0	99 (97–100)	39
QUANT_CI	95	0	100 (100–100)	48
QUANT_CI	99	0	100 (100–100)	40
S‐CAL	80	100	26 (8–50)	−11
S‐CAL	90	100	26 (8–50)	−17
S‐CAL	95	100	26 (8–50)	−23
S‐CAL	99	100	26 (8–51)	−39
SALτ	80	85	45 (2–94)	−9
SALτ	90	63	55 (2–100)	3
SALτ	95	59	59 (2–100)	1
SALτ	99	55	62 (2–100)	−15
TM_QBCB	80	1	97 (88–100)	42
TM_QBCB	90	0	99 (97–100)	50
TM_QBCB	95	0	100 (100–100)	52
TM_QBCB	99	0	100 (100–100)	40

## 4 Discussion

In this article, we analyzed the performance of a wide range of stopping methods on their ability to reliably determine the optimal point to stop screening in a ranked set of abstracts. The results show that almost all stopping methods cannot be used without a high risk of missing relevant records, and no method is able to reliably achieve optimal work savings that are theoretically possible for a given recall target. This highlights a trade‐off between having relative certainty about achieving a recall target and optimal work savings.

When evaluating the utility of stopping methods, we argue that a method is unreliable and cannot be used safely when the functionality depends on fine‐tuning hyperparameters or on an optimal ranking. In a real‐world screening scenario, reviewers do not have perfect knowledge to retrospectively choose the best hyperparameters or distinguish an imperfect ranking from actual convergence, which would indicate that all relevant records were found, and it is safe to stop annotating abstracts. To this end, we focus less on reporting aggregated performance metrics but present the entire distribution of results across a wide range of data sets, repeated ranking simulations, and hyperparameter settings. In the same way, we argue that metrics such as work‐saved‐over‐sampling can be misleading when judging the effectiveness of stopping methods, as this is also influenced by the ability of the ranking method to find relevant records based on prior include/exclude decisions. Therefore, we focus on the deviation from the theoretically optimal stopping point on a given inclusion curve. However, this requires setting recall targets for target‐unaware methods as well, with the choice of these targets impacting our evaluation metrics.

Aligning results from such a wide range of data sets means that we have to choose a common frame of reference. This has the side effect that some implications are hidden. For example, the interpretation of a reported percentage of 50% unused work‐saving potential is vastly different if the recall target is reached after screening 10% or 90% of a data set. Particularly for larger data sets, this can substantially impact the person‐hours required to complete a systematic evidence synthesis.

As discussed above, we found that the choice of ranking models strongly influenced the potential to save work. Furthermore, even if a ranking method is able to find all relevant records early on in the process, the shape of the inclusion curve toward that point can have a strong impact on the performance of stopping methods. Particularly, plateaus from local minima are often wrongly interpreted as a signal to stop, and some methods use window sizes or other smoothing strategies to mitigate this. The best way to mitigate is to screen a large enough random sample in the beginning. We chose a fixed size for the initial sample based on anecdotal evidence. Future work is required to systematically explore adaptive mechanisms to reduce that size based on data set characteristics to switch to prioritized screening as late as needed but as soon as possible. The introduced uncertainties by stopping early and the apparent need for a large enough training data set to train a good ranking model—one that is able to identify relevant records well—imply that priority screening with early stopping should only be used in larger reviews. For this analysis, we chose 1000 to be the lower bound to ensure high statistical significance of all results.

Using classifier scores to rank unseen records may pose issues that require further research. In doing so, we need to make the assumption that slight differences in scores actually carry some meaning with regard to how relevant a record is compared to another one, given prior screening decisions. However, the scores of classification models might describe the distance to a decision boundary, feature similarity to previously seen records, or others. The score distribution of some models is also tightly grouped around zero (or negative one) and one, and allows no true fine‐grained ranking. If all scores only vary in the seventh decimal and are close to zero as the inclusion curve starts to converge, it is not clear how that ranking actually differs from random sampling at that point. Similarly, after each retraining of the model as the inclusion curve converges, there always tend to be a few records with higher scores that reveal a few more relevant records that might not have been found at that point without retraining. This suggests that the frequency of retraining might also have an impact on the utility of a ranking to determine a safe point to stop screening.

Stopping methods cannot be used without a clear and reliable guide on how to choose hyperparameters in an ongoing priority screening project without full knowledge. Based on our wide range of tested hyperparameters, we were not able to derive reproducible rules of thumb to determine safe and reliable settings. With an increasing number of technology‐assisted reviews that use automation in some or all steps of the process, uncertainties have a compounding effect on the final outcomes. For example, an imperfect screening recall implies some records might be missed, and their reported effect sizes will not be included in the final meta‐analysis. Being able to estimate how many records are affected allows the authors to adjust the reported confidence interval. Researchers developing stopping methods should therefore focus on uncertainty‐aware methods to enable an estimation of those compounding effects. Furthermore, the wider research community and users of evidence need to form a common understanding of which uncertainties are acceptable in which context.

Prior work in this field uses a range of different definitions of what a “good” stopping method is and in what context it is used. For example, for technology assisted reviews (TAR) in the legal domain, it seems to be good enough to aim for 70% or lower recall targets. Our results clearly show that we can not simply apply such methods in the context of systematic reviews with requirements for high target recalls and a high certainty of meeting set targets. Given the range of digital evidence synthesis tools offering priority screening capabilities, it is crucial to form a shared understanding of the expectations for a “good” stopping method. Our framework not only helps to simplify future research on improved stopping methods, it also introduces a standardized way for robust evaluations and a shared, yet extensible way to measure “success.” We urge tool developers to integrate robust stopping methods in their priority screening tools and educate users on how to safely apply those in their synthesis pipeline. At the same time, we encourage authors to transparently report how they used stopping methods in their systematic maps or reviews.

## 5 Conclusion

Safe use of AI in evidence synthesis projects requires rigorous evaluation to ensure robust results. Priority screening is a widely adopted method to reduce time for identifying relevant articles for a systematic map or review. However, it is crucial to combine machine‐learning‐based ranking with statistically sound stopping methods.

The modular architecture of our open‐source evaluation framework makes it easy to add stopping methods without tightly integrating them into framework utilities, so they can also easily be used elsewhere by tool developers or users of digital evidence synthesis tools. The framework serves as a good foundation for future benchmarking across a growing set of reference data sets, stopping methods, and ranking models. At the moment, some existing stopping methods are not yet part of the framework and adding larger fully‐screened data sets from a wide range of different research areas for evaluation would be desirable. Future work could also use a variety of ranking mechanisms and randomly sample the data used for their initialization to evaluate the performance and proper calibration of the uncertainty evaluation of stopping methods across an even larger set of possible contexts.

Our empirical evaluation of 81 real‐world data sets reveals that almost all existing stopping methods are not fit‐for‐purpose. In our experiments, only one method (CMH) has not missed any relevant records and provided work savings. QUANT_CI and TM_QBCB have met the recall target in most experiments, but also missed relvant records in some cases. This comes at the expense of stopping very conservatively, which, to an extent, can relatively safely be mitigated by lowering the confidence level. Another method has a similar overshoot characteristic but misses up to half the relevant records in a few cases. Future work is needed to develop safe and robust stopping methods that can reliably save work by stopping early at a set recall target and confidence level.

## Author Contributions

**Tim Repke:** conceptualization, investigation, methodology, software, resources, project administration, formal analysis, validation, visualization, writing – original draft, writing – review and editing, data curation. **Francesca Tinsdeall:** conceptualization, investigation, writing – original draft, writing – review and editing, validation, methodology, software, formal analysis, data curation. **Diana Danilenko:** conceptualization, data curation, methodology, investigation, validation, writing–review and editing. **Sergio Graziosi:** data curation, investigation, resources, validation, writing – review and editing. **Finn Müller‐Hansen:** writing – review and editing, formal analysis, validation, software, investigation. **Lena Schmidt:** conceptualization, investigation, writing – review and editing, methodology, software. **James Thomas:** data curation, funding acquisition, resources, writing – review and editing. **Gert van Valkenhoef:** writing – review and editing, formal analysis.

## Ethics Statement

The authors have nothing to report.

## Consent

The authors have nothing to report.

## Conflicts of Interest

Tim Repke, Sergio Graziosi, and James Thomas are developers of popular screening and review platforms (NACSOS, EPPI‐reviewer). Finn Müller‐Hansen is one of the two co‐authors of CMH (also known as the CMH method [20]). The other authors declare no conflicts of interest.

## Supporting information

Supplementary Information

## Data Availability

The code and raw simulation data are available at https://doi.org/10.5281/zenodo.17497378 and on GitHub at https://github.com/destiny-evidence/stopping-methods. Unfortunately, we are not able to share the fully annotated data, including abstracts, due to copyright restrictions.
