# Supplementary material for: Don't Stop Me Now, `Cause I'm Having a Good Time Screening: Evaluation of Stopping Methods for Safe Use of Priority Screening in Systematic Reviews
Source: Cochrane Evid Synth Methods. 2026 Jan 21;4(1):e70068. doi: 10.1002/cesm.70068 (PMC12825451; doi:10.1002/cesm.70068)
Supplement: Supplementary file 1 — Supplementary Information [file CESM-4-e70068-s001.pdf]

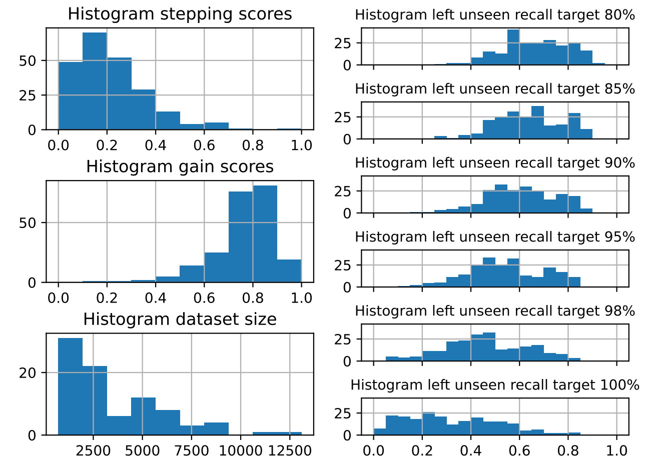

FIGURE A1 Histograms of where different theoretical recall targets are reached

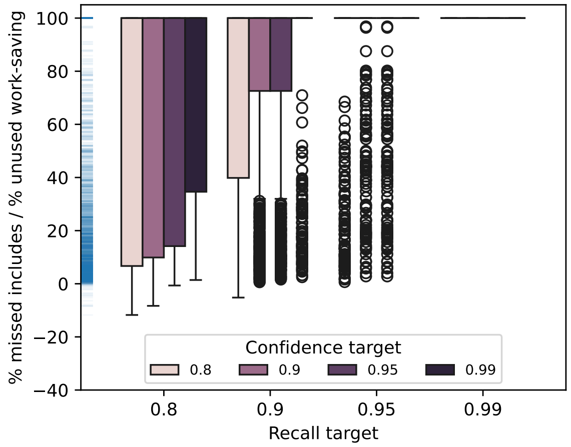

FIGURE A2 Impact on performance for TM_QBCB showing relation of confidence target and recall target.

How to cite this article: Repke, T., Tinsdeall, F., Danilenko, D., Flemyng, E., Graziosi, S., Müller-Hansen, F., Schmidt, L., Thomas, J. and Valkenhoef, G.. Don't stop me now, 'cause I'm having a good time screening: Evaluation of stopping methods for safe use of priority screening in systematic reviews *Cochrane Evidence Synthesis and Methods* 2025;00(00):1–18.

APPENDIX

A EXTENDED RESULTS SECTION

In Table A1 and Table A2 we list the distribution of the x/y-axis histograms presented in Figure 2. With Figure A6 we also present an alternative grid of scatter-plots that shows the relationship between the proportion of missed relevant records and distribution of dataset a ranking simulations (Figure A1)

B STOPPING METHOD PARAMETERS

In this section we list all tested hyper-parameter settings for each stopping method. Where available, we use the same set of recall targets (80%, 90%, 95%, 99%) and confidence levels (80%, 90%, 95%, 99%) for each method. Additionally, we use all combinations of

- APRIORI: recall targets, inclusion threshold $\in \{0.25, 0.5, 0.75, 0.9\}$
- BATCHPRECISION: batch size $\in \{500, 1000, 2000\}$, threshold$\in \{0.05, 0.1, 0.2\}$
- CMH: recall targets, confidence levels
- CURVE_FITTING: recall targets, confidence levels, windows$\in \{10, 50\}$
- HEURISTIC_FIX: consecutive includes$\in \{50, 100, 200, 300\}$
- HEURISTIC_FRAC: proportional consecutive includes$\in \{1\%, 5\%, 7.5\%, 10\%, 20\%\}$
- HEURISTIC_RAND: recall targets

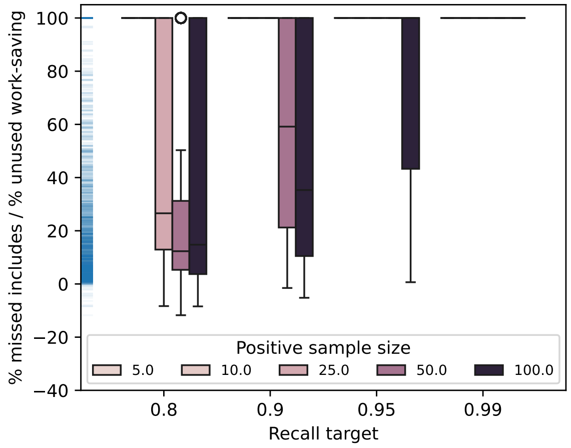

FIGURE A3 Impact of method hyper-parameters on overall result for TM_QBCB showing relation of number of positive samples and recall target.

- HEURISTIC_SCORES: recall targets, threshold$\in \{0.05, 0.1, 0.2\}$
- KNEE: window size for smoothing$\in \{500\}$, polynomial$\in \{1\}$, threshold for slope ratio$\in \{2, 3, 4, 7\}$, threshold for distance to diagonal $\in \{0.2, 0.3, 0.4\}$
- METHOD2399: alpha$\in \{1.0, 1.1, 1.2\}$
- QUANT_CI: recall targets, confidence interval scaling$\in \{1, 2\}$
- IPP: recall targets, confidence levels, number of windows$\in \{5, 10, 20, 50, 100\}$
- SAL$_\tau$: recall targets, confidence levels, alpha$\in \{0.1, 0.3, 0.5, 0.8, 1.0\}$, with or without normalising the space
- S-CAL: recall targets, bias$\in \{0.9, 1.0, 1.05, 1.1, 1.2, 1.3\}$, sample size$\in \{0.5, 0.8, 0.9\}$

TABLE A1 Distribution of values shown in x-axes of Figure 2. Bins per row are aggregated top to bottom (e.g. first row aggregates for the range 94–100% missed relevant records, last row aggregates everything above 94% unused work-saving potential).

APRIORI	BATCHPRECISION	CMH	CURVE_FITTING	HEURISTIC_FIX	HEURISTIC_FRAC	HEURISTIC_RANDOM	HEURISTIC_SCORES	IPP	KNEE	METHOD2399	QUANT_CI	S-CAL	SALτ	TM_QBCB	% missed includes / % unused work-saving
0.0	0.1	0.0	0.2	57.0	58.7	0.0	23.5	8.4	2.1	0.0	0.0	0.4	16.7	0.0	-100
0.0	6.5	0.0	2.4	5.5	3.9	0.0	23.2	14.9	3.9	0.0	0.0	22.6	18.5	0.0	-94
0.0	5.7	0.0	2.9	2.0	1.6	0.3	14.8	9.3	2.5	0.1	0.0	15.3	5.0	0.0	-84
0.1	9.7	0.0	3.6	1.5	1.2	1.8	10.7	6.4	2.8	2.4	0.0	17.2	5.4	0.0	-73
0.6	8.6	0.0	3.2	1.7	0.8	2.6	7.0	4.9	3.6	3.7	0.0	16.1	1.7	0.0	-63
1.0	9.8	0.0	3.6	3.0	1.2	3.6	4.6	5.0	4.5	2.7	0.0	13.2	10.2	0.0	-52
1.0	8.1	0.0	3.2	3.8	2.8	5.6	3.4	4.3	5.9	7.0	0.0	4.4	2.5	0.0	-42
0.9	7.4	0.0	3.0	5.2	5.0	8.2	1.9	3.1	7.3	5.4	0.0	4.8	8.1	0.0	-31
0.5	5.8	0.0	2.2	5.0	5.2	14.2	1.2	2.3	7.1	6.1	0.2	1.1	0.0	0.0	-21
0.4	4.3	0.2	1.5	4.0	4.1	12.7	1.0	1.4	7.4	4.6	0.6	0.7	0.0	0.1	-10
0.3	2.7	0.0	0.5	1.0	0.5	16.6	0.7	1.2	4.2	2.2	4.8	0.1	2.1	7.5	0
0.0	3.2	5.8	0.1	1.9	3.0	3.0	0.2	0.9	4.6	1.2	7.3	0.1	0.0	4.3	10
0.0	2.4	19.7	0.0	1.5	2.3	1.5	0.1	1.3	4.5	2.8	6.8	0.0	0.0	2.4	21
0.0	2.6	16.3	0.2	1.7	2.1	0.8	0.0	1.7	3.2	3.1	4.8	0.0	0.0	1.3	31
0.0	1.9	13.5	0.6	0.6	1.3	0.4	0.0	1.4	3.3	1.6	3.6	0.0	0.0	0.9	42
0.0	1.8	11.3	1.3	0.9	1.3	0.3	0.1	0.9	2.6	1.9	4.6	0.0	1.0	0.5	52
0.0	1.6	9.2	1.7	0.6	0.7	0.1	0.0	1.2	3.1	0.1	3.7	0.0	0.0	0.4	63
0.0	1.5	9.9	2.0	0.3	0.9	0.2	0.0	1.3	2.0	2.2	2.9	0.0	0.0	0.3	73
0.0	0.9	7.2	1.8	0.4	0.5	0.1	0.0	1.0	1.9	1.9	4.3	0.0	0.0	0.1	84
95.3	15.1	6.8	65.8	2.5	3.0	27.9	7.7	29.2	23.4	50.5	56.4	3.8	28.7	82.1	94

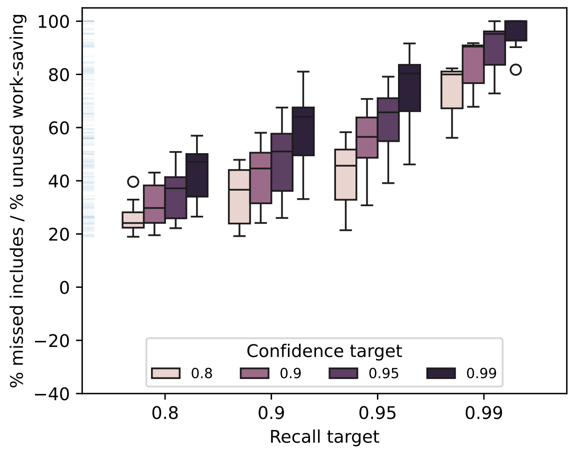

FIGURE A4 Impact of method hyper-parameters on overall result for CMH showing relation of confidence and recall targets.

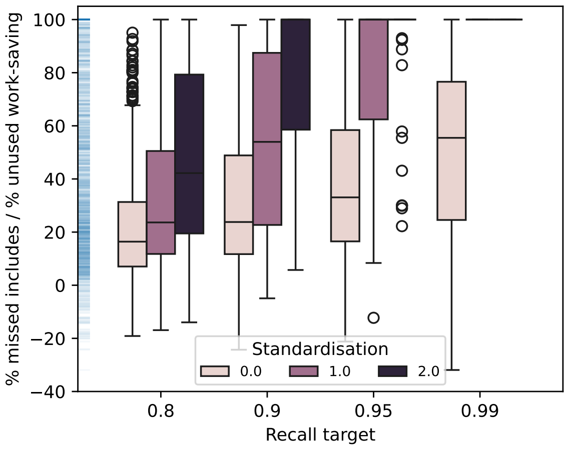

FIGURE A5 Impact of method hyper-parameters on overall result for QUANT_CI showing relation of n-std and recall targets.

- TM_QBCB: recall targets, confidence levels, positive sample size $\in \{5, 10, 25, 50, 100\}$

TABLE A2 Distribution of values shown in y-axes of Figure 2. Aggregation bins are top-to-bottom, where the first row shows the percentage of simulations per method that had 0–650 records unseen when the stopping rule was met.

APRIORI	BATCHPRECISION	CMH	CURVE_FITTING	HEURISTIC_FIX	HEURISTIC_FRAC	HEURISTIC_RANDOM	HEURISTIC_SCORES	IPP	KNEE	METHOD2399	QUANT_CI	S-CAL	SAL$_{\tau}$	TM_QBCB	Remaining after recall target
20.4	44.8	13.6	19.1	44.8	44.8	20.4	20.4	20.4	44.8	44.8	20.4	20.3	18.8	18.8	0
26.7	26.0	36.4	23.4	26.0	26.0	26.7	26.7	26.7	26.0	26.0	26.7	25.5	43.8	25.1	650
14.9	5.8	6.8	15.9	5.8	5.8	14.9	14.9	14.9	5.8	5.8	14.9	15.3	18.8	16.5	1300
5.2	8.5	4.5	6.6	8.5	8.5	5.2	5.2	5.2	8.5	8.5	5.2	5.5	0.0	6.2	1950
6.8	4.5	9.1	7.9	4.5	4.5	6.8	6.8	6.8	4.5	4.5	6.8	7.3	6.2	7.1	2600
8.0	4.9	6.8	9.2	4.9	4.9	8.0	8.0	8.0	4.9	4.9	8.0	7.8	12.5	8.1	3250
5.2	1.3	6.8	5.2	1.3	1.3	5.2	5.2	5.2	1.3	1.3	5.2	5.0	0.0	5.0	3900
3.9	1.8	6.8	3.9	1.8	1.8	3.9	3.9	3.9	1.8	1.8	3.9	3.9	0.0	4.0	4550
2.1	1.3	2.3	1.2	1.3	1.3	2.1	2.1	2.1	1.3	1.3	2.1	1.8	0.0	1.3	5200
0.7	0.4	0.0	0.5	0.4	0.4	0.7	0.7	0.7	0.4	0.4	0.7	0.7	0.0	0.7	5850
1.2	0.0	2.3	1.0	0.0	0.0	1.2	1.2	1.2	0.0	0.0	1.2	1.4	0.0	1.5	6500
2.4	0.4	2.3	2.3	0.4	0.4	2.4	2.4	2.4	0.4	0.4	2.4	2.6	0.0	2.5	7150
1.1	0.0	2.3	1.4	0.0	0.0	1.1	1.1	1.1	0.0	0.0	1.1	1.2	0.0	1.3	7800
0.3	0.0	0.0	0.5	0.0	0.0	0.3	0.3	0.3	0.0	0.0	0.3	0.4	0.0	0.4	8450
0.1	0.0	0.0	0.2	0.0	0.0	0.1	0.1	0.1	0.0	0.0	0.1	0.1	0.0	0.1	9100
0.1	0.0	0.0	0.2	0.0	0.0	0.1	0.1	0.1	0.0	0.0	0.1	0.1	0.0	0.1	9750
0.2	0.0	0.0	0.4	0.0	0.0	0.2	0.2	0.2	0.0	0.0	0.2	0.2	0.0	0.3	10400
0.4	0.0	0.0	0.7	0.0	0.0	0.4	0.4	0.4	0.0	0.0	0.4	0.5	0.0	0.6	11050
0.2	0.0	0.0	0.4	0.0	0.0	0.2	0.2	0.2	0.0	0.0	0.2	0.2	0.0	0.3	11700

TABLE A3 Distribution of values shown in y-axes of Figure A6. Aggregation bins are top-to-bottom similar to Table A1

APRIORI	BATCHPRECISION	CMH	CURVE_FITTING	HEURISTIC_FIX	HEURISTIC_FRAC	HEURISTIC_RANDOM	HEURISTIC_SCORES	IPP	KNEE	METHOD2399	QUANT_CI	S-CAL	SAL$_{\tau}$	TM_QBCB	Recall when stopped
0.0	0.0	0.0	0.0	0.0	0.0	0.0	0.0	0.0	0.0	0.0	0.0	0.0	0.0	0.0	-10
0.0	0.1	0.0	0.1	55.9	55.7	0.0	23.7	12.1	1.3	0.0	0.0	0.5	16.7	0.0	-3
0.0	3.4	0.0	3.5	5.6	6.5	0.0	30.7	15.1	4.4	0.0	0.0	10.3	8.3	0.0	2
0.0	5.2	0.0	4.8	2.4	1.8	0.0	11.2	4.7	2.5	0.0	0.0	18.2	10.2	0.0	8
0.1	4.1	0.0	2.3	1.3	0.9	0.0	6.4	5.1	1.1	0.0	0.0	15.2	1.7	0.0	14
0.3	3.5	0.0	2.5	0.6	0.4	0.0	2.5	3.5	0.2	0.0	0.0	14.2	0.0	0.0	20
0.3	3.1	0.0	2.6	0.0	0.1	0.3	2.0	4.4	0.4	0.0	0.0	10.5	3.5	0.0	26
0.5	2.0	0.0	2.4	0.2	0.2	0.8	1.1	3.5	0.0	0.0	0.0	7.7	1.0	0.0	32
1.0	2.4	0.0	1.3	0.0	0.0	0.3	1.4	1.9	0.0	0.0	0.0	6.0	2.5	0.0	38
0.5	2.4	0.0	1.4	0.0	0.0	0.8	1.8	1.6	0.0	0.0	0.0	7.8	0.0	0.0	44
0.5	1.0	0.0	0.9	0.0	0.0	1.7	1.8	1.7	0.0	0.0	0.0	2.5	0.0	0.0	50
0.4	1.1	0.0	1.2	0.0	0.0	2.8	1.7	1.2	0.0	0.0	0.0	4.0	0.8	0.0	56
0.3	1.0	0.0	0.9	0.0	0.0	4.5	1.7	1.2	0.0	0.0	0.0	2.9	0.8	0.0	62
0.2	0.3	0.0	1.1	0.0	0.0	7.4	1.5	1.1	0.0	0.0	0.3	0.0	0.8	0.0	68
0.1	1.3	0.0	0.3	0.1	0.1	8.6	1.5	0.8	0.0	0.0	0.7	0.0	15.0	0.1	74
0.1	2.6	0.0	0.6	0.1	0.2	12.8	1.0	0.7	0.1	0.9	1.2	0.0	1.7	1.1	80
0.1	5.1	0.0	0.3	1.0	0.6	13.5	0.8	1.1	1.6	0.9	4.7	0.0	0.0	3.9	86
0.2	15.5	12.5	0.6	7.8	5.3	14.1	1.2	1.9	15.2	13.5	15.9	0.0	2.5	7.1	92
95.3	45.7	87.5	73.3	24.9	28.3	32.4	8.0	38.3	73.0	84.8	77.2	0.0	34.4	87.7	98

TABLE A4 Datasets used in the analysis selected from 380 datasets in CLEF, SYNERGY, SIGIR, and from EPPI-reviewer

Dataset	Records (total)	Records (relevant)	Records (% relevant)	Dataset	Records (total)	Records (relevant)	Records (% relevant)
CLEF: CD005139	4564	105	2.3	CLEF: CD012179	8376	297	3.5
CLEF: CD007394	2375	90	3.8	CLEF: CD012347	1093	16	1.5
CLEF: CD007427	1497	115	7.7	CLEF: CD012599	6617	527	8.0
CLEF: CD007431	1921	20	1.0	CLEF: CD012661	3344	192	5.7
CLEF: CD008054	3023	265	8.8	CLEF: CD012669	1155	70	6.1
CLEF: CD008122	1762	237	13.5	SYNERGY: Leenaars_2020	7216	583	8.1
CLEF: CD008691	1254	71	5.7	SYNERGY: Muthu_2021	2719	336	12.4
CLEF: CD008803	4692	99	2.1	SYNERGY: Smid_2020	2627	27	1.0
CLEF: CD008874	1798	114	6.3	SYNERGY: van_der_Waal_2022	1970	33	1.7
CLEF: CD008892	1276	64	5.0	EPPI: Heart 0503	5554	47	0.8
CLEF: CD009020	1468	130	8.9	EPPI: Heart 0809	3087	61	2.0
CLEF: CD009069	1665	78	4.7	EPPI: Heart 0901	1328	56	4.2
CLEF: CD009175	4862	61	1.3	EPPI: Heart 0904	2157	108	5.0
CLEF: CD009185	1279	90	7.0	EPPI: Heart 1002	1115	48	4.3
CLEF: CD009323	3631	86	2.4	EPPI: Heart 1022	1338	38	2.8
CLEF: CD009372	2054	24	1.2	EPPI: Heart 1103	1430	27	1.9
CLEF: CD009519	4805	104	2.2	EPPI: Heart 1109	2517	55	2.2
CLEF: CD009551	1879	46	2.4	EPPI: Heart 1118	1189	30	2.5
CLEF: CD009579	4795	128	2.7	EPPI: Heart 1120	1507	64	4.2
CLEF: CD009642	1869	62	3.3	EPPI: Heart 1205	1390	22	1.6
CLEF: CD009647	2641	52	2.0	EPPI: Heart 1211	1076	29	2.7
CLEF: CD009925	5276	420	8.0	EPPI: Heart 1306	2155	189	8.8
CLEF: CD009944	1000	114	11.4	EPPI: Heart 1312	3873	129	3.3
CLEF: CD010276	4671	51	1.1	EPPI: Heart 1406	2315	110	4.8
CLEF: CD010296	4491	53	1.2	EPPI: Kidney3.2	3168	75	2.4
CLEF: CD010438	2364	36	1.5	EPPI: Kidney_lowerproteinuria	6267	316	5.0
CLEF: CD010502	2602	141	5.4	EPPI: NICE CG121	5874	299	5.1
CLEF: CD010632	1486	32	2.2	EPPI: NICE CG132	2537	284	11.2
CLEF: CD010657	1685	137	8.1	EPPI: NICE CG170	1240	38	3.1
CLEF: CD010753	2248	28	1.2	EPPI: NICE Ian Saunders	5288	1957	37.0
CLEF: CD010864	2204	39	1.8	EPPI: VTEPOCTandDDimer	3573	177	5.0
CLEF: CD011134	1798	189	10.5	EPPI: Vaccines2QualScreening	9098	476	5.2
CLEF: CD011145	10787	202	1.9	EPPI: automation	13095	236	1.8
CLEF: CD011431	1123	280	24.9	EPPI: neonatal1.3&5.3	1255	71	5.7
CLEF: CD011548	9262	102	1.1	EPPI: neonatal5.1&5.2	7016	188	2.7
CLEF: CD011787	3842	110	2.9	EPPI: projectChildInvolvement	4997	313	6.3
CLEF: CD011975	6743	552	8.2	EPPI: projectCovidBarn	2799	108	3.9
CLEF: CD011984	6746	411	6.1	EPPI: review-1253	6676	126	1.9
CLEF: CD012010	5758	178	3.1	EPPI: review-1453	3629	75	2.1
CLEF: CD012069	2903	283	9.7	EPPI: shareddecisionmaking1.1	6897	151	2.2

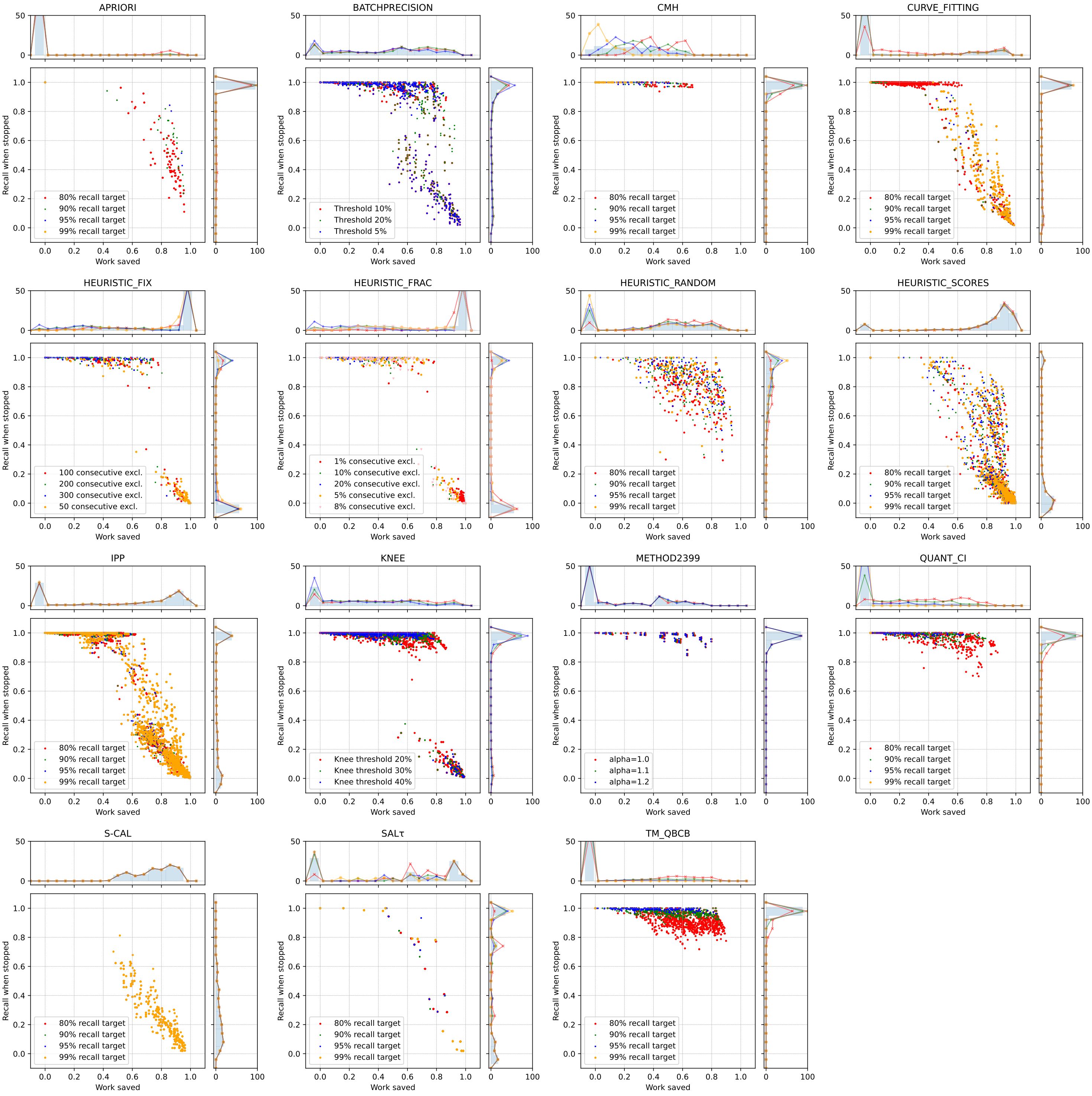

FIGURE A6 Distribution of trade-off between work saved thanks to early stopping and missed relevant records. The ideal stopping method would have all points at (0, 0), meaning that no relevant records were missed and the stopping rule invoked at the theoretically perfect time with no additional work. Results are grouped by stopping method and respectively one key hyper-parameter. The histograms help to reveal the distribution of points for each axis which is otherwise obfuscated due to over-plotting in the scatterplot. Histogram lines correspond to points of each hyper-parameter grouping whereas bars show the overall distribution.
